# Infectious Proctitis Mimicking Advanced Rectal Cancer: A Case Report and Update on the Differential Diagnosis of Rectal Ulcerations

**DOI:** 10.3390/jcm14155254

**Published:** 2025-07-24

**Authors:** Anca Maria Pop, Roman Zimmermann, Szilveszter Pekardi, Michela Cipriani, Angelika Izabela Gajur, Diana Moser, Eva Markert, Alexander Kueres-Wiese

**Affiliations:** 1 Department of Internal Medicine, HOCH Health Ostschweiz, Wil Hospital, 9500 Wil, Switzerland; 2 Department of Gastroenterology and Hepatology, HOCH Health Ostschweiz, Wil Hospital, 9500 Wil, Switzerland; roman.zimmermann@h-och.ch (R.Z.); szilveszter.pekardi@h-och.ch (S.P.); alexander.kueres-wiese@h-och.ch (A.K.-W.); 3 Department of Infectious Diseases, Infection Prevention and Travel Medicine, HOCH Health Ostschweiz, Cantonal Hospital St. Gallen, 9007 St. Gallen, Switzerland; michela.cipriani@h-och.ch; 4 Department of Radiology and Nuclear Medicine, HOCH Health Ostschweiz, Wil Hospital, 9500 Wil, Switzerland; angelika.gajur@h-och.ch; 5 Institute for Pathology, HOCH Health Ostschweiz, Cantonal Hospital St. Gallen, 9007 St. Gallen, Switzerland; diana.moser@h-och.ch (D.M.); eva.markert@h-och.ch (E.M.)

**Keywords:** proctitis, syphilis, sexually transmitted diseases, rectal ulcer

## Abstract

**Background**: Infectious proctitis remains an underrecognized entity, although sexually transmitted diseases, especially bacterial infections, exhibit a marked increase in their incidence. **Methods**: Here, we report a case of a 44-year-old man who presented to the emergency department with lower abdominal and rectal pain, tenesmus, fever and night sweats for the past 6 days. **Results**: The computed tomography initially revealed a high suspicion of metastatic rectal cancer. The endoscopic findings showed a 5 cm rectal mass, suggestive of malignancy. The histologic examination showed, however, no signs of malignancy and lacked the classical features of an inflammatory bowel disease, so an infectious proctitis was further suspected. The patient reported to have had unprotected receptive anal intercourse, was tested positive for Treponema pallidum serology and received three doses of intramuscular benzathine penicillin G. A control rectosigmoidoscopy, imaging at 3 months and histological evaluation after antibiotic treatment showed a complete resolution of inflammation. **Conclusions**: Syphilitic proctitis may mimic various conditions such as rectal cancer or inflammatory bowel disease and requires a high degree of suspicion. Clinicians need to be aware of infectious proctitis in high-risk populations, while an appropriate thorough medical history may guide the initial diagnostic steps.

## 1. Introduction

Rectal ulcerations are uncommon and underrecognized manifestations of sexually transmitted diseases (STDs) due to their non-pathognomonic clinical features and inconclusive histopathological findings [1]. However, STDs must be considered in every patient presenting with rectal symptoms, due to the major implications regarding treatment and prognosis [2]. Furthermore, according to the Global Burden of Diseases 1990–2019 analyses, STDs reported a consistent increase in their absolute incidence [3].

During the last decade, in the light of emerging effective HIV treatments, the control of STDs has reached a plateau or may have even worsened for diseases such as syphilis [3,4]. The most commonly transmitted anorectal pathogens are represented by *Neisseria gonorrhoeae*, *Chlamydia trachomatis*, *Treponema pallidum* and Herpes simplex virus (HSV), out of which *T. pallidum* reported the most marked increase since 1993 [3,5]. This trend is emphasized in higher income countries with functional screening programs, where there has been a significant rise in the incidence of bacterial infections, namely a 28% increase in gonorrhea and a 74% increase in syphilis, as reported in 2021 in the United States [4]. Syphilis exhibits a markedly high prevalence of up to 7.5% in men who have sex with men (MSM) [6]. Moreover, there are significant synergistic associations in the case of a HIV and syphilis co-infection; HIV weakens the immune system, predisposing to syphilis acquisition while syphilis fragilizes the rectal mucosa making it more vulnerable to HIV acquisition [7]. Infectious proctitis may be asymptomatic in some gonococcal and chlamydia infections [2], or may present as multiple ulcers mimicking an inflammatory bowel disease (IBD) or even a malignant tumour [8,9]. Based on these aspects, the initial acquisition of a broad personal history is of utmost importance before guiding the first diagnostic steps.

We present a case of syphilitic proctitis in a young man firstly confounded with an advanced rectal carcinoma, highlighting the most important features in the diagnosis of infectious proctitis. Moreover, the aim of our paper is to suggest a possible diagnostic work-up for proctitis and to summarize the most relevant aspects in the differential diagnosis of rectal ulcerations.

## 2. Case Presentation

A 44-year-old male patient presented to the Emergency Department of our hospital, complaining of a 6-day history of abdominal pain with migratory localisation and tenesmus. Moreover, the patient reported accompanying flu-like symptoms such as fever and malaise as well as night sweats. On the day of presentation, the abdominal pain was mainly localised in the left lower quadrant. The stools were normally formed and coloured; however, they exhibited a high frequency, up to hourly, and were painful. Weight loss was denied by the patient. The medical history was insignificant, revealing only well-controlled asthma. The family history was negative for malignancy as well as for IBD. Upon clinical examination, the patient reported tenderness on palpation in the left lower abdominal quadrant. Vital signs were normal with a blood pressure of 125/79 mmHg, heart rate of 95/min and an oxygen saturation of 97% on room air.

**Figure 1:**
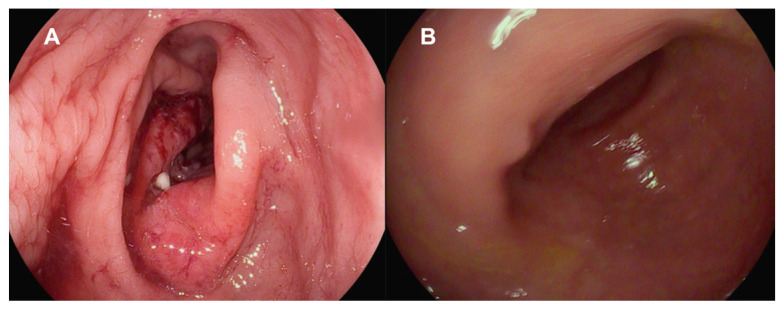
(**A**) Semicircular, ulcerating mass at the rectosigmoidal junction with irregular margins, consistent with a neoplastic process, without signs of perforation or obstruction. (**B**) Complete resolution of inflammation after antibiotic treatment.

**Figure 2:**
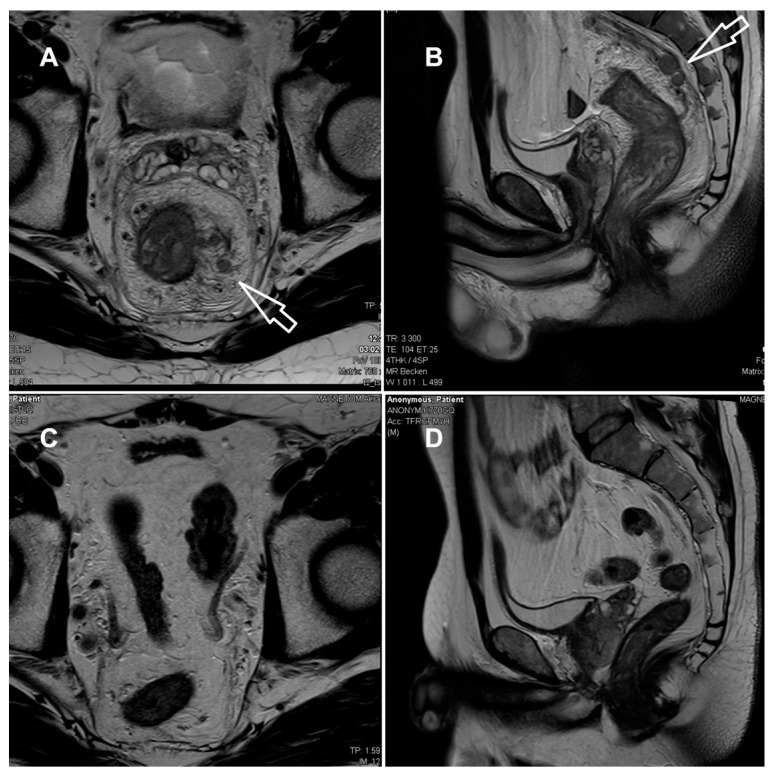
MRI of the rectum at presentation (**A**,**B**) and after 3 months (**C**,**D**). (**A**,**B**) T2-weighted axial (**A**) and sagittal (**B**) at initial presentation, showing long-segment, circumferential thickening of the rectal wall (white arrows) with marked mural irregularities, edema, perineural and perivascular infiltration; multiple lymph nodes with irregular shape within and beyond the mesorectal fascia, extending to the external iliac region. Infiltration of the mesorectal fascia and peritoneum with mucosal diffusion restriction. Mild pelvic ascites. Suspected colorectal carcinoma staged as T4a N2b M1 MRF+ EMVI+. (**C**,**D**) After antibiotic treatment, complete regression of the rectal wall and mesorectal fascia thickening; regression of edema and no lymphadenopathy. (MRF = mesorectal fascia, EMVI = extramural vascular invasion).

**Figure 3:**
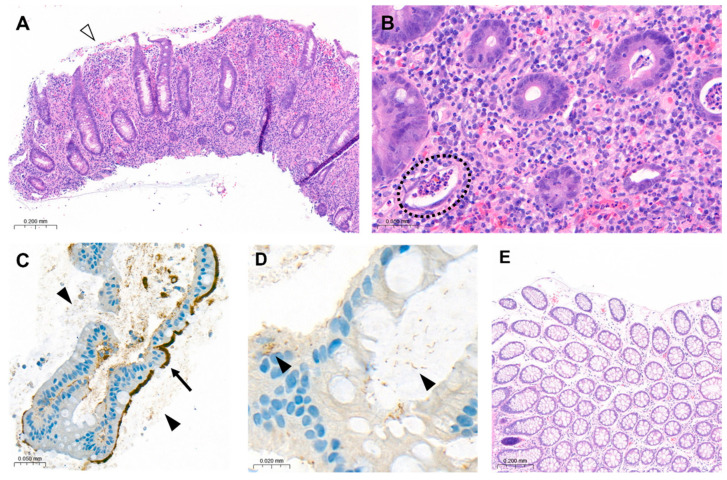
Histology of rectal biopsies taken before (**A**–**D**) and after (**E**) antibiotic treatment. (**A**) Colon mucosa with increased chronic active inflammation in the lamina propria, erosions (white arrow) and preserved architecture. (**B**) Cryptitis and crypt abscesses (black circle). (**C**,**D**) Immunohistochemistry for *T. pallidum* highlights superficially distributed spirochetic bacteria on the mucosal surface (arrow) and in crypt lumina (black arrowhead). (**E**) Unremarkable colon mucosa post antibiotic treatment. H&E hematoxylin and eosin.

The laboratory parameters showed signs of inflammation with an elevated C-reactive protein of 110 mg/L; a leukocyte count of 4.3 G/L was normal. The computed tomography (CT) scan revealed a high suspicion of rectal carcinoma in the middle and distal third of the rectum with multiple regional and non-regional lymph nodes metastases. The patient was subsequently admitted to hospital and scheduled for a coloscopy with biopsy on the following day. The coloscopy showed a 5 cm tumour in the distal rectum with unremarkable surrounding mucosa in the rest of the colon and terminal ileum (Figure 1A). A magnetic resonance imaging (MRI) of the pelvis was advised for better definition and further described a semi-circumferential rectal carcinoma with vascular invasion, lymph nodes metastases and possible peritoneal involvement (Figure 2A,B). A CT scan of the thorax was unremarkable. The histological evaluation of the mucosal biopsies showed features of acute inflammation with erosions, cryptic abscesses with focal distribution and crypt destruction; in the lamina propria, there was an increased chronic lymphoplasmacytic inflammatory infiltrate with granulation tissue and epitheloid cell granulomas. There was no architectural disorder or signs of malignancy (Figure 3A,B). The Wartin–Starry stain and *T. pallidum* immunohistochemistry (IHC) (Figure 3C,D) highlighted multiple spirochetic bacteria on the mucosal surface in a band-like distribution, without clear evidence of intraepithelial bacteria, as characteristic of *T. pallidum* infection.

After clinicopathologic correlation of the available investigations, further serologic testing of the patient was performed. The patient reported a history of multiple sexual male partners. Tests for HIV and hepatitis B and C were negative. Both treponemal-specific tests (*T. pallidum*-hemagglutination-assay = TPHA and *T. pallidum*-antibodies) and a nontreponemal test (rapid plasma reagin, RPR) were positive. The polymerase chain reaction (PCR) results of a rectal swab were negative for *T. pallidum*, *N. gonorrhoeae*, *C. trachomatis* and *HSV 1/2*. Due to the negative *T. pallidum*-PCR from the rectal swab and lack of previous syphilis testing, which could have helped to estimate the time of infection, we diagnosed a late latent syphilis. Therefore, a treatment with a total of three doses of 2.4 million units of intramuscular benzathine penicillin G (once a week for 3 weeks) was recommended. The patient was symptom-free after the completion of the antibiotic treatment and no Jarisch–Herxheimer reaction was observed. A control rectosigmoidoscopy (Figure 1B), as well as the MRI (Figure 2C,D) at 3 months and the histologic evaluation (Figure 3E) showed a complete resolution of the inflammation.

## 3. Discussion

Syphilitic proctitis is a rare condition, which requires a high degree of suspicion before diagnosis and can otherwise mimic multiple conditions, extending from IBD to malignant patterns. Our case emphasizes the importance of a broad clinical history before initiating diagnostic steps, usually difficult to be performed in an emergency department. Despite the increasing incidence of STDs, especially of bacterial infections, the infectious proctitis seems to remain underrecognized. Firstly, rectal STD screening among MSM is less frequently performed compared to urethral screening [10]. Previous research showed that sexual health providers were two to six times more likely to perform urethral STD testing for gonorrhea and chlamydia than rectal testing [11]. Since the diagnosis of another STD is one of the most significant predictors of HIV acquisition and asymptomatic carriage is possible, testing for urethral, pharyngeal and rectal STDs should simultaneously be performed [12]. The clinical presentation is in the great majority of cases identical in STD and IBD proctitis, as symptoms such as anal discharge, tenesmus, fever, vomiting or weight loss significantly overlap [13]. Endoscopically, STD proctitis is associated with friable, ulcerated and hyperemic mucosa, which is also encountered in IBD. Moreover, the presence of rectal strictures or masses may indicate signs of malignancy [14,15]. Therefore, the diagnosis is often suspected based on the histologic examination, which fails to identify malignancy or IBD features. Histologically, the STD colitis lacks the IBD features such as cryptic damage, mucosal eosinophilia or presence of granulomas, being characterized by an abundant submucosal and perivascular plasma cell infiltrate [16].

To the best of our knowledge, there are approximately 50 cases of syphilitic proctitis described beginning from the 1960s. A summary of the available cases in the literature published in the last 25 years and their clinical and histological features is presented in Table 1.

**Figure 4:**
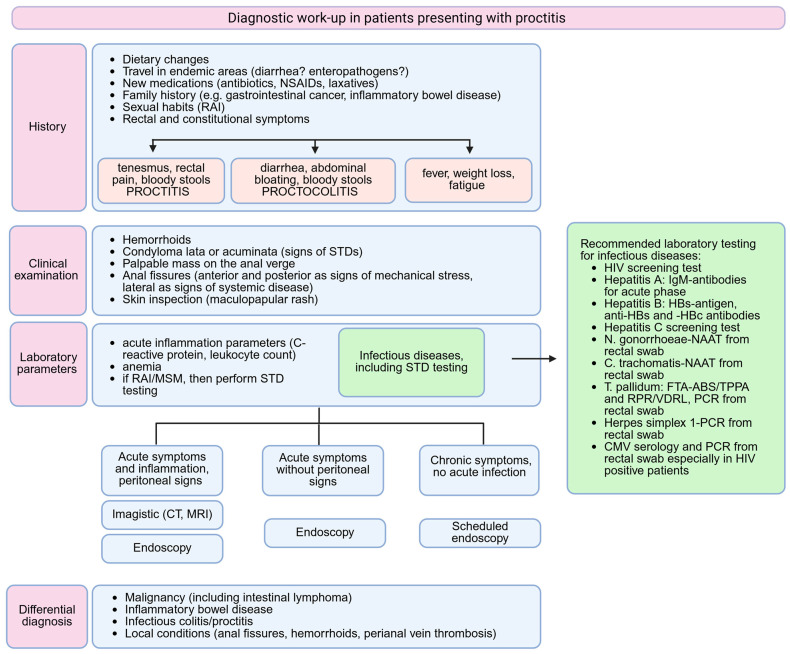
Proposed diagnostic algorithm in patients presenting with rectal symptoms. Created in BioRender. Pop, A. (2025) https://BioRender.com/ohvs81s. (CMV = cytomegalovirus, CT = computed tomography, FTA-ABS = fluorescent treponemal antibody absorption, HIV= human immunodeficiency virus, MRI = magnetic resonance imaging, MSM = men who have sex with men, NAAT = nucleic acid amplification test, NSAIDs = nonsteroidal anti-inflammatory drugs, PCR = polymerase chain reaction, RAI = receptive anal intercourse, RPR = rapid plasma reagin, STD = sexually transmitted diseases, TPPA = Treponema pallidum particle agglutination, VDRL = Venereal Disease Research Laboratory).

Based on the previously published clinical data on STD proctitis, we suggest a possible algorithm, which can guide a reasonable diagnosis in patients presenting with rectal symptoms (Figure 4).

All cases were diagnosed in men and transgender women, initially presenting with symptoms and clinical findings related to rectal malignancy. A first diagnosis of infectious proctitis sparing an elaborate imagistic and histologic work-up was encountered only in cases of HIV-positive MSM or in patients presenting directly to a sexual health service with chronic symptoms [30,31,32,33,34,37].

A thorough medical history before initiating further endoscopic and imagistic diagnosis is important, as the identification of risk factors such as MSM, receptive anal intercourse (RAI) in the last 6 months or HIV seropositive status warrants STD testing [2]. If patients omit or refuse to mention these aspects, then a complete clinical examination could identify the presence of condylomata or anal fissures. Condylomata may sometimes be misdiagnosed as haemorrhoids and in patients presenting with multiple rectal and systemic symptoms a simple diagnosis of haemorrhoids should be doubted [38]. The number and localization of anal fissures matters, as the vast majority of anal fissures are due to mechanic stress, being in 90% of cases located in the posterior midline [49]. The presence of multiple or lateral fissures may indicate systemic disease such as syphilis, HIV, malignancy, Crohn’s disease or tuberculosis [49,50]. An acute presentation with new onset symptoms (fever, severe anal and abdominal pain, vomiting) can pose difficulties in following a specific algorithm, by necessitating an immediate diagnosis. An imagistic scan is not usually recommended as a first-line investigation, as it may lead to false diagnosis, such as rectal malignancy [51]. However, a CT scan may be performed when peritoneal signs are present, in a case in which an endoscopy is contraindicated, or in order to evaluate for possible internally draining abscess, strictures or mucosal ulcerations in the more proximal intestine [1]. Due to its higher costs, MRI is not a standard tool in an emergency department and is therefore performed in a later setting.

When infectious proctitis is suspected, several pathogens must be taken into consideration and simultaneously tested for identifying co-infections. The most prevalent rectal pathogens in MSM are *C. trachomatis* (average prevalence 9%) and *N. gonorrhoeae* (average prevalence 6.1%), which are both in the majority of cases asymptomatic; appropriate testing relies on the detection of pathogens by nucleic acid amplification tests (NAATs) performed from rectal swabs [52]. Lymphogranuloma venereum, caused by the *C. trachomatis* genovars L1, L2 and L3, is an endemic infection in MSM and usually presents as proctitis with purulent anal discharge, rectal bleeding and pain with possible pelvic lymphadenopathy; the detection of specific *C. trachomatis*-DNA is diagnostic and should be performed in all MSM with positive *C. trachomatis* rectal swabs [53]. Testing for rectal *M. genitalium* is not routinely recommended in MSM, since it has not been significantly associated with the development of proctitis and should be considered only in cases with persistent anal symptoms after excluding other infectious causes [2]. Cytomegalovirus can also cause rectal ulcerations and must always be tested in HIV-positive patients, as it is considered an indicator of advanced disease [2].

A combination of directly identified pathogens on the histologic sample and a positive serologic test is the preferred method for diagnosing an infectious proctitis [54]. In our case, there was no direct evidence of *T. pallidum* bacteria on the rectal biopsy; however, the lack of malignant and IBD classic features together with the serologic confirmation of an active syphilis indicated the diagnosis of a syphilitic proctitis. A diagnosis of primary rectal syphilis is frequently difficult as the rectal chancre, characteristic of this stage, is painless, superficial and discharges clear secretion. On the other hand, syphilitic proctitis indicates the progression to a secondary syphilis, being accompanied by systemic manifestations like fever or generalized lymphadenopathy [54].

The actual guidelines recommend the use of treponemal screening tests such as TPHA, *T. pallidum* particle agglutination assay (TPPA) or enzyme immunoassay (EIA), which are sensitive in detecting early syphilis, but may give false positive results. Nontreponemal tests such as RPR or the Venereal Disease Research Laboratory (VDRL) can detect only active syphilis and can miss very early forms of disease. If screening is based on the performance of both a treponemal and nontreponemal test, then the nontreponemal test must be performed quantitatively [54]. It is important to mention that in some patients a negative nontreponemal test may be followed by a positive treponemal test. This is due to an excess of antibodies in the undiluted serum, referred to as the prozone phenomenon in the early stages of syphilis, and is more often encountered in HIV-positive patients [34,39]. In our medical centre, however, a treponemal test is always first performed, and is, when positive, followed by a nontreponemal test. This allows the detection of the disease in early stages and reduces the chance of false negative interpretations due to the prozone phenomenon.

PCR tests are recommended for atypical sites of syphilis, such as oral cavity or rectum, where a distinction from the commensal spirochetes is needed [55]. However, the sensitivity and specificity of PCR in the secondary and latent stages of syphilis remain low, which impairs PCR testing from becoming a routine diagnostic tool. In a study by Shields et al., the sensitivity of PCR swabs in secondary syphilis was 50%, while the specificity was 100% [56]. Another study conducted by Costa-Silva et al. reported a sensitivity of 81% with the same high specificity of 100% [57]. The high chance of false negative PCR swabs in secondary syphilis was partially explained by various research groups based on the following possible hypotheses: the sample collection in routine PCRs usually lacks the required quality for a correct acquisition and the high antibody titres encountered in secondary syphilis impair the development of a high treponemal DNA load in tissue samples [58,59,60]. The direct visualization of spirochetes using dark-field microscopy or Warthin–Starry staining is specific, but difficult to be performed and often provides a false negative result [54]. When serology is negative, the IHC staining has excellent specificity in diagnosing secondary syphilis, but is not part of the routine diagnosis [58].

In our case, the sexual history, lack of malignant features on the histologic sample and the positive *T. pallidum* serology were considered suggestive and enough for sustaining the diagnosis of syphilitic proctitis, which was later confirmed by the resolution of symptoms under appropriate antibiotic therapy.

## 4. Conclusions

Syphilitic proctitis, although relatively rare among causes of infectious proctitis, requires a high degree of suspicion in order to avoid clinical overdiagnosis. It may mimic various conditions from IBD to rectal malignancy. Therefore, clinicians must be aware of the increasing incidence of STDs and perform the appropriate screening tests in high-risk populations.

## Figures and Tables

**Table 1:** Cases of syphilitic proctitis published in the scientific literature.

No.	Author, Year	Gender, Age	Clinical Presentation	Imagistic Findings	Endoscopic Findings	Clinical Diagnosis	Histology	Laboratory Findings
1	Hunter, 2025 [15]	Male, 60, MSM	Perianal lesions, anorectal pain, painful defecation, bright red blood with bowel movements 2 cm ulcerated nodule in the left lateral perianal skin	N/A	Due to patient discomfort not performed	Haemorrhoids, suspected HPV and LGV lesions	Ulcerated nodule: moderate perivascular and interstitial lymphocytic infiltrate, plasma cells, neutrophils and numerous eosinophils. Low-grade squamous intraepithelial lesion. *T. pallidum:* IHC staining positive	*T. pallidum:* TPPA and RPR positive, rectal swab PCR positive HIV negative
2	Yin, 2024 [17]	Male, 29 MSM	Hematochezia and anal pain 4 cm-mass away from anus	MRI: rectal wall thickening, inguinal and mesenteric lymphadenopathy	Nodular, irregular lesions with scattered ulcers and hemorrhage	Rectal carcinoma	Lymphoplasmacytic infiltrates, no signs of neoplasia, no granulomas *T. pallidum*: Warthin–Starry silver staining positive	Not mentioned
3	Cantu Lopez, 2024 [18]	TGW, 49	Left abdominal pain, non-bloody emesis, weight loss, constipation with mucous	CT: rectal wall thickening, perirectal adenopathy	Erythema, inflammation, thickened rectal folds with near luminal obstruction	Lymphoma, colitis	Expanded lamina propria, neutrophilic and lymphoplasmacytic infiltrate *T. pallidum:* IHC staining positive	*T. pallidum:* RPR/FTA-ABS positive HIV negative
4	Afzal, 2024 [19]	Male, 64 MSM	Significant change in bowel habits, palpable rectal mass	CT: mild rectal wall thickening of the rectum, local lymphadenopathy; MRI: normal	10-mm elevated rectal lesion with central ulceration, 5–6 cm from the anal verge	Rectal carcinoma	Non-specific active chronic inflammation *T. pallidum:* Steiner staining positive	*T. pallidum:* EIA and RPR positive HIV negative
5	Bae, 2024 [20]	Male, 23	Right-sided inguinal mass, tenderness in the right inguinal area	CT: inguinal, mesorectal and presacral adenopathy, 10 cm-long circumferential rectal wall thickening	Edematous and hyperemic mucosa, rectal wall thickening	Rectal lymphoma	Dense infiltration of polymorphic lymphoid cells and histiocytes in the lamina propria with ulcers, increased numbers of plasma cells and eosinophils	*T. pallidum:* RPR/FTA-ABS positive HIV negative
6	Ranabhotu, 2023 [21]	Male, 72	Rectal bleeding and pain	N/A	Firm perianal mass	Anal carcinoma	High-grade squamous intraepithelial lesion, consistent with HPV infection, intraepithelial neutrophils and abundant submucosal plasma cells *T. pallidum:* IHC staining positive	*T. pallidum:* RPR/FTA-ABS positive HIV negative
7	Peine, 2023 [22]	Male, 38 MSM	Two weeks of obstipation and abdominal pain	CT: large bowel obstruction by a 7 cm × 6.8 cm rectal mass MRI: mesorectal fascia involvement	Benign-appearing rectal stricture at 2 cm from the anal verge	Rectal carcinoma	Severe active proctitis, no evidence for malignancy *T. pallidum*: Warthin–Starry silver staining positive	*T. pallidum:* RPR positive *N. gonorrhoeae* PCR: positive HIV positive
8	Alcantara, 2023 [23]	Male, 35 MSM	Rectal bleeding, tenesmus	N/A	Rectal ulcers with clean bases and raised edges	N/A	Lymphoplasmacytic infiltrate in the lamina propria, cryptitis and cryptic microabscesses *T. pallidum*: Warthin–Starry silver staining positive	*T. pallidum:* RPR/FTA-ABS positive *C. trachomatis*: IgM and IgG positive HIV positive,
9	Mansilla, 2023 [24]	Male, 40	Rectal ulceration	Rectal lesion with mesenteric and extra mesenteric adenopathy	Ulcerated rectal vegetating lesion	Rectal carcinoma	Non-specific polymorphous inflammation	*T. pallidum:* serology positive HIV positive
10	Smith, 2022 [25]	Male, 39 MSM	Right upper quadrant pain, tenesmus and diarrhoea	CT: short irregular thickening of the rectal wall, mesorectal adenopathy	2-cm ulcer with heaped margins and a necrotic base in the distal rectum	Metastatic rectal cancer	Ulceration with chronic inflammation, atypical crypt epithelium, no evident malignant changes	*T. pallidum:* RPR positive HIV positive
11	Cain, 2022 [26]	Female, 46	Rectal bleeding	N/A	1-cm-submucosal mass inside the anal verge	Carcinoid or gastrointestinal stromal tumour	Small lymphocytes infiltrating lamina propria	*T. pallidum:* antibody test, EIA and RPR negative
12	Costales, 2021 [27]	Male, 32, MSM	Lower abdominal and rectal pain, diarrhoea with hematochezia for 2 weeks	CT: distal sigmoid and rectal wall thickening, perirectal, pelvic, retroperitoneal lymphadenopathy	Rectal non-bleeding ulcers, friable ulcerated mucosa	STD colitis, later rectal malignancy	Focal ulceration, non-caseating granulomas, multinucleated giant cells, no signs of malignancy *T. pallidum*: Warthin–Starry silver staining and IHC staining negative	*T. pallidum:* RPR and TPPA positive
13	Ahmed, 2020 [28]	Male, 59 MSM	Fever, rectal pain	PET/CT: intense metabolic activity in the rectum, porta hepatis and internal mammary lymph nodes	5-cm-rectal mass	Lymphoproliferative process	Ulceration and infiltration by large atypical lymphoid cells, concomitant diagnosis of lymphoma and syphilitic proctitis *T. pallidum*: IHC staining negative	*T. pallidum:* RPR positive HIV positive
14	Patil, 2020 [29]	Male, 66, MSM	Constipation, abdominal pain	CT: diffuse rectal mucosal thickening, perirectal fat stranding, mesorectal adenopathy	Two lesions in the distal rectum, edema and erythema	Rectal carcinoma	Chronic lymphoid and neutrophil inflammatory infiltrate, mucosal ulceration and granulation tissue, no malignancy *T. pallidum*: Warthin–Starry silver staining negative	*T. pallidum:* TPPA, RPR and IgM positive HIV positive
15	Siddiqui, 2020 [30]	Male, 25, MSM	Rectal bleeding, pain, tenesmus, fatigue, weight loss, no night sweats or fever	N/A	Severe ulcerative proctitis, anal fissure	STD or IBD	Active proctitis and lymphocytic inflammation *T. pallidum*: IHC staining positive	*T. pallidum:* RPR positive *C. trachomatis:* rectal swab PCR positive HIV negative
16	Kumar, 2019 [31]	Male, 41 MSM	6-month-anal pain, no rectal discharge, perianal ulceration	N/A	N/A	STD	N/A	*T. pallidum:* VDRL positive *C. trachomatis*: PCR positive HIV negative
17	Sousa, 2019 [8]	Male, 66 MSM	Rectal bleeding, mucoid discharge, proctalgia and fever	CT: concentric thickening of the distal rectum, densification of the mesorectal fat	Irregularity, edema, hyperemia and mucus in the distal rectal mucosa	N/A	Inconclusive	*T. pallidum:* RPR and antibody test positive *C. trachomatis:* IgM positive ∙ Rectal swabs inconclusive
18	Teng, 2018 [32]	Male, 47, MSM	Rectal discharge and bleeding, tenesmus	N/A	Multiple irregular and friable ulcerations	N/A	Nonspecific ulcer and scattered Epstein–Barr virus–positive lymphoid cells *T. pallidum*: IHC staining positive	*T. pallidum:* VDRL and TPHA positive HIV and hepatitis B positive
19	Lopez, 2018 [33]	Male, 48	Rectal bleeding, tenesmus, popular erythematous rash on the trunk and extremities, inguinal lymph nodes	N/A	Serpiginous ulcers with erythematous and edematous surrounding mucosa	N/A	Acute inflammatory cells spirochetes staining positive	*T. pallidum:* serology positive HIV negative
20	Alcantara, 2018 [34]	Male, 53, MSM	Rectal bleeding, pain and tenesmus, penis ulcers, inguinal adenopathy	N/A	Ulcer covering 70% of the rectal circumference in the distal rectum, well defined and firm edges, ulcer base covered with mucus	N/A	Severe inflammatory lymphomononuclear infiltrate, granulomas *T. pallidum*: Warthin–Starry silver staining positive	*T. pallidum:* VDRL non-reactive, FTA-ABS positive HIV positive
21	Allan, 2018 [35]	Male, 50	No symptoms (routine screening)	N/A	Rectal polyp, nodular areas in the distal rectum (routine screening colonoscopy)	N/A	Chronic lymphoplasmacytic infiltrate in lamina propria *T. pallidum*: IHC staining positive	*T. pallidum:* RPR and TPPA positive HIV positive
22	Serigado, 2018 [36]	Male, 47, MSM	Rectal bleeding, fatigue, decreased appetite, abdominal pain	CT: left lateral wall thickening in the distal rectum, hypodense hepatic lesions, splenomegaly	One 3-cm, firm, raised and centrally ulcerated mass at the anorectal junction, with irregular borders and bleeding	Rectal carcinoma, Kaposi sarcoma, STD	Granulomatous inflammation, no malignancy *Spirochetes:* IHC staining positive	*T. pallidum:* VDRL and FTA-ABS positive HIV positive
23	Diaz, 2017 [37]	Male, 35 MSM	Two-week history of intermittent bloody stools	N/A	Irregular rectal ulcer with a fibrinous surface and friable mucosa	N/A	Chronic inflammatory infiltrate of plasma cells, severe cryptitis, no neoplastic cells *T. pallidum*: Warthin–Starry silver staining positive	*T. pallidum:* RPR and TPHA positive HIV positive
24	Zeidman, 2016 [38]	Male, 33, MSM	Rectal bleeding	N/A	mucosal inflammation of the distal rectum, patchy erythema and edema	Crohn’s disease	Lamina propria expansion and separation of the colonic crypts by a histiocyte-rich infiltrate with foci of lymphoid hyperplasia *T. pallidum*: IHC staining positive	*T. pallidum:* RPR and antibody test positive HIV negative
25	Gopal, 2015 [39]	Male, 52 MSM	Anal canal ulcer	N/A	N/A		Chronic plasma cell-rich infiltrate between the squamous epithelium and lamina propria, poorly formed granulomas *T. pallidum*: IHC staining positive	*T. pallidum:* RPR nonreactive, FTA-ABS reactive, PCR perianal swab positive HIV positive
26	Gopal, 2015 [39]	Male, 44	Nonhealing anal canal ulcer	N/A	N/A		Chronic plasma cell-rich infiltrate between the squamous epithelium and lamina propria *T. pallidum*: IHC staining positive	*T. pallidum:* RPR and FTA-ABS positive HIV unknown
27	Gopal, 2015 [39]	Male, 51	Anal canal ulcer	N/A	N/A		Ulcer with chronic plasma cell-rich infiltrate between the squamous epithelium and lamina propria, poorly formed granulomas *T. pallidum*: IHC staining positive	*T. pallidum:* RPR and TPPA positive HIV unknown
28	Gopal, 2015 [39]	Male, 31 MSM	Anal canal mass and rectal bleeding	N/A	N/A		Granulation tissue with plasma cells, mild cryptitis *T. pallidum*: IHC staining positive	*T. pallidum:* RPR and antibody test positive HIV positive
29	Cerreti, 2015 [40]	Male, 48 MSM	Proctorrhagia Palpable mass 5 cm away from anus	MRI: thickening of the rectal wall, infiltration of the mesorectal fat, lymph nodes in the perirectal fat	Single ulcer with regular edges and lunate shape, occupying one-third of the rectal circumference	Rectal carcinoma	No evidence of malignancy *T. pallidum*: Warthin–Starry silver staining positive	*T. pallidum*: serology positive HIV positive
30	Bensusan, 2014 [41]	Male, 50, MSM	Rectal bleeding, frequent stools	CT: reduction in the rectal caliber, locoregional, abdominal, and inguinal adenopathy	Rectal ulcer with elevated and smooth edges and fibrinous surface	STD	Active ulcer, no malignancy *Spirochetes:* IHC staining negative	*T. pallidum*: RPR positive HIV negative
31	Yilmaz, 2011 [42]	Male, 38, MSM	Rectal pain Palpable rectal mass	CT: unremarkable	Hard, ulcerative lesion in distal rectum	Crohn’s disease	Chronic inflammatory plasma-cell infiltrate, superficial necrosis, fibropurulent exudate, granulomatous inflammatory crypt destruction	*T. pallidum*: VDRL and FTA/ABS positive HIV negative
32	Milligan,2010 [43]	Male, 51 MSM	Rectal pain and bleeding Palpable rectal mass	MRI: malignancy features	Findings suggesting rectal malignancy	Rectal carcinoma	Granulation tissue and marked inflammatory infiltrate, no evidence of malignancy *T. pallidum*: identified on n dark-field examination	Genito-urinary screening positive for *T. pallidum*
33	Cha, 2010 [44]	Male, 45, MSM	Anal pain, tenesmus, bloody stools, mucus discharge, inguinal adenopathy	CT: irregular rectal wall thickening, adenopathy MRI: rectal wall thickening, perirectal fat infiltration	3×4 cm well-demarcated, deep ulcer on the lower rectum extending from the anal canal	Rectal carcinoma	Diffuse chronic plasma cell inflammatory infiltrate, no neoplastic changes *T. pallidum*: Warthin–Starry silver staining positive	*T. pallidum*: RPR and TPPA positive
34	Zhao, 2010 [45]	Male, 51	Anorectal discomfort, tenesmus, mucous discharge and bloody stools, weight loss	CT: local inhomogeneous rectal wall thickening 3 cm from the anal verge	Irregular ulcerated mass, hyperemia and erosion of the rectal wall	Rectal carcinoma	Extensive lymphocytic, plasmocytic and neutrophilic infiltrate, lymphoid follicles, ulceration, but no heterotypic cells or lymph epithelial lesion *T. pallidum*: IHC staining not performed	*T. pallidum*: TPPA positive Hepatitis B serology positive HIV negative
35	Furman, 2008 [46]	Male, 28	Rectal pain	N/A	Ulcerative proctitis	NA	Chronic active colitis with cryptitis and ulceration *T. pallidum:* Steiner staining positive	*T. pallidum*: RPR and FTA-ABS positive HIV positive
36	Song, 2005 [47]	Male, 30, MSM	Rectal pain and bleeding, tenesmus, inguinal adenopathy	N/A	Two indurated masses of ca. 2 cm in the middle and lower rectum with ulcerated and depressed surface	Rectal carcinoma	Lymphoid hyperplasia without evidence of malignancy *T. pallidum*: IHC staining not performed	*T. pallidum*: VDRL, FTA-ABS positive HIV negative
37	Chan, 2003 [48]	Male, 32, MSM	Rectal discharge, macular rash on the soles, inguinal lymphadenopathy	N/A	N/A	N/A	N/A	*T. pallidum*: RPR and TPHA positive HIV positive, *C. trachomatis* and *N. gonorrhoeae* positive

CT = computed tomography, EIA = enzyme immunoassay, FTA-ABS = fluorescent treponemal antibody absorption, IBD = inflammatory bowel disease, IHC = immunohistochemistry, HIV = human immunodeficiency virus, HPV = human papilloma virus, LGV = Lymphogranuloma venereum, MRI = magnetic resonance imaging, MSM = men who have sex with men, N/A = not applicable, PCR = polymerase chain reaction, RPR = rapid plasma regain, STD = sexually transmitted diseases, TGW = transgender woman, TPHA= *T. pallidum*-hemagglutination-assay, TPPA = *T. pallidum*-particle agglutination, VDRL = Venereal Disease Research Laboratory

## Data Availability

The original contributions presented in this study are included in the article. Further inquiries can be directed to the corresponding author.
